# A New Meroditerpene and a New Tryptoquivaline Analog from the Algicolous Fungus *Neosartorya takakii* KUFC 7898

**DOI:** 10.3390/md13063776

**Published:** 2015-06-15

**Authors:** War War May Zin, Suradet Buttachon, Jamrearn Buaruang, Luís Gales, José A. Pereira, Madalena M. M. Pinto, Artur M. S. Silva, Anake Kijjoa

**Affiliations:** 1 ICBAS—Instituto de Ciências Biomédicas Abel Salazar, Universidade do Porto, Rua de Jorge Viterbo Ferreira, 228, 4050-313 Porto, Portugal; E-Mails: wwmzin.chem.yu@gmail.com (W.W.M.Z.); nokrari_209@hotmail.com (S.B.); lgales@ibmc.up.pt (L.G.); jpereira@icbas.up.pt (J.A.P.); 2 Interdisciplinary Centre of Marine and Environmental Research (CIIMAR), Rua dos Bragas 289, 4050-313 Porto, Portugal; E-Mail: madalena@ff.up.pt; 3 Division of Environmental Science, Faculty of Science, Ramkhamhaeng University, Bangkok 10240, Thailand; E-Mail: jbuaruang@ru.mail.go.th; 4 Instituto de Biologia Molecular e Celular (IBMC), Universidade do Porto, Rua de Jorge Viterbo Ferreira, 228, 4050-313 Porto, Portugal; 5 Laboratório de Química Orgânica, Departamento de Ciências Químicas, Faculdade de Farmácia, Universidade do Porto, Rua de Jorge Viterbo Ferreira, 228, 4050-313 Porto, Portugal; 6 Departamento de Química & QOPNA, Universidade de Aveiro, 3810-193 Aveiro, Portugal; E-Mail: artur.silva@ua.pt

**Keywords:** *Neosartorya takakii*, meroditerpene, sartorenol, tryptoquivaline U, aszonapyrone A, chevalone B, aszonalenin, 6-hydroxymellein

## Abstract

A new meroditerpene sartorenol (**1**), a new natural product takakiamide (**2**) and a new tryptoquivaline analog (**3**) were isolated, together with nine known compounds, including aszonapyrone A, chevalone B, aszonalenin, acetylaszonalenin, 3′-(4-oxoquinazolin-3-yl) spiro[1*H*-indole-3,5′-oxolane]-2,2′-dione, tryptoquivalines L, F and H, and the isocoumarin derivative, 6-hydroxymellein, from the ethyl acetate extract of the culture of the algicolous fungus *Neosartorya takakii* KUFC 7898. The structures of the new compounds were established based on 1D and 2D NMR spectral analysis, and, in the case of sartorenol (**1**) and tryptoquivaline U (**3**), X-ray analysis was used to confirm their structures and to determine the absolute configuration of their stereogenic carbons. Compounds **1**, **2** and **3** were evaluated for their antimicrobial activity against Gram-positive and Gram-negative bacteria, and multidrug-resistant isolates from the environment; however, none exhibited antibacterial activity (MIC ˃ 256 mg/mL). The three new compounds did not show any quorum sensing inhibition in the screening protocol based on the pigment production by *Chromobacterium violaceum* (ATCC 31532).

## 1. Introduction

In recent years, marine-derived fungi have been demonstrated to be a rich and promising source of novel anticancer, antibacterial, antiplasmodial, anti-inflammatory, and antiviral agents [1]. To date, more than one thousand unique molecular structures have been discovered from marine-derived fungi. Several reviews on marine fungi [2,3,4] have shown that a variety of secondary metabolites isolated from marine-derived fungi had not been produced by terrestrial fungi, and these metabolites possibly act as a chemical defense, enabling marine-derived fungi to survive competition with native microorganisms [5]. Thus, marine-derived fungi, which successfully fostered their armamentarium against bacterial competitors for millions of years, can be considered as a potential source of antibiotics.

**Figure 1:**
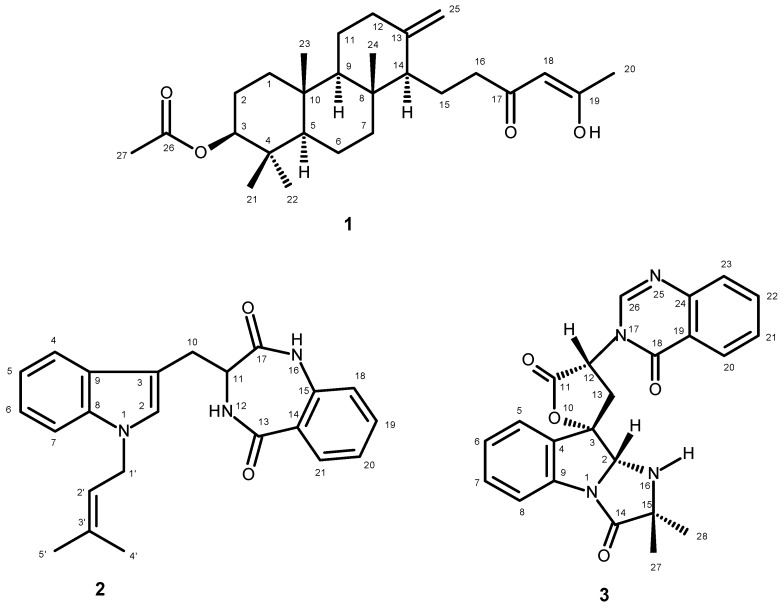
New secondary metabolites isolated from the ethyl acetate extract of the culture of *N. takakii* KUFC 7898.

In our ongoing pursuit of new natural products with antibacterial activity produced by marine-derived fungi of the genera *Neosartorya* and *Aspergillus*, we have investigated the secondary metabolites of a Thai collection of *Neosartorya takakii* KUFC 7898, isolated from the marine macroalga *Amphiroa* sp., collected from Samaesarn Island in the Gulf of Thailand. The ethyl acetate extract of its culture yielded, in addition to the previously reported aszonapyrone A [6], chevalone B [7,8], aszonalenin [6], acetylaszonalenin [6], 3′-(4-oxoquinazolin-3-yl) spiro[1*H*-indole-3,5′-oxolane]-2,2′-dione [9], tryptoquivalines L, F and H [9], and 6-hydroxymellein [10], three new compounds including a meroditerpene sartorenol (**1**), a prenylated indole alkaloid takakiamide (**2**), and a new tryptoquivaline analog, which we have named tryptoquivaline U (**3**) (Figure 1). Compounds **1**–**3** were screened for their antibacterial activity against Gram-positive and Gram-negative bacteria, and multidrug-resistant isolates from the environment as well as for their quorum sensing inhibitory activity.

## 2. Results and Discussion

**Figure 2:**
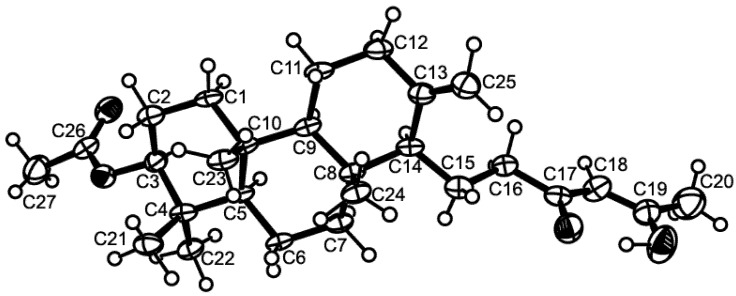
Ortep view of sartorenol (**1**).

Compound **1** was isolated as white crystals (mp, 122–123 °C) and its molecular formula C_27_H_42_O_4_ was established on the basis of the (+)-HRESIMS *m*/*z* 431.3175 [M + H]^+^ (calculated 431.3161), indicating seven degrees of unsaturation. The IR spectrum showed absorption bands for hydroxyl (3393 cm^−1^), conjugated ketone carbonyl (1645 cm^−1^), ester carbonyl (1728 cm^−1^), and olefin (1558, 1540 cm^−1^) groups. The ^13^C NMR, DEPT and HSQC spectra (Table 1, Supplementary Figures S3 and S4) exhibited the signals of one conjugated ketone carbonyl (δ_C_ 194.7), one ester carbonyl (δ_C_ 171.0), two quaternary sp^2^ (δ_C_ 147.7 and 191.1), one methine sp^2^ (δ_C_ 99.9), one methylene sp^2^ (δ_C_ 106.4), three quaternary sp^3^ (δ_C_ 37.4, 37.8, 39.8), one oxymethine sp^3^ (δ_C_ 80.8), three methine sp^3^ (δ_C_ 55.4, 56.4 and 59.8), eight methylene sp^3^ (δ_C_ 18.7, 19.6, 23.3, 23.6, 37.2, 38.0, 38.2 and 40.5) and six methyl (δ_C_ 15.3, 16.3, 16.4, 21.3, 24.9 and 28.0) carbons. The ^1^H NMR spectrum (Table 1, Supplementary Figure S1) revealed the presence of one hydrogen-bonded hydroxyl group of an enol at δ_H_ 15.47, s, two exocyclic methylene protons at δ_H_ 4.84, brs and 4.50, brs, one olefinic proton at δ_H_ 5.45, s, and the protons of six methyl groups at δ_H_ 0.69, s, 0.83, s, 0.84, s, 0.86, s, 2.05, s (integrating for two methyls). Except for the enolic hydroxyl group, the olefinic proton and the conjugated ketone carbonyl (δ_C_ 194.7), the ^1^H and ^13^C data (Table 1, Supplementary Figures S1 and S3) revealed the presence of a perhydrophenanthrene moiety, similar to that of aszonapyrone A [6]. Like aszonapyrone A, the acetoxyl group on C-3 of compound **1** was β, as was evidenced by the coupling constants of H-3 (δ_H_ 4.48, dd, *J* = 10.9, 4.6 Hz). Another portion of the molecule, which consists of C_6_H_9_O_2_, was identified as (4*Z*)-5-hydroxy-3-oxohex-4-enyl group due to the HMBC correlations (Table 1, Supplementary Figure S5) of H_3_-20 (δ_H_ 2.05, s) to C-18 (δ_C_ 99.9) and C-19 (δ_C_ 191.1), of H-18 (δ_H_ 5.45, s) to C-16 (δ_C_ 37.2), C-17 (δ_C_ 194.7), C-19 (δ_C_ 191.1) and C-20 (δ_C_ 24.9), as well as the NOESY correlation (Supplementary Figure S6) between H-18 and H_3_-20. That C-15 of the (4*Z*)-5-hydroxy-3-oxohex-4-enyl group was connected to C-14 of the perhydrophenanthrene moiety was supported by the correlations between H-14 (δ_H_ 1.59, m) and H-15 (δ_H_1.86, m) in the COSY spectrum (Table 1, Supplementary Figure S2), as well as by the HMBC correlation of H_2_-15 to C-13 (δ_C_ 147.7). The structure and stereochemistry of compound **1** were unambiguously determined by X-ray analysis (Figure 2), and the absolute configurations of C-3, C-5, C-8, C-9, C-10 and C-14 were identified as 3*S*, 5*R*, 8*R*, 9*R*, 10*R* and 14*S*, respectively. Since **1** is a new compound, we have named it sartorenol.

**Table 1:** ^1^H and ^13^C NMR (CDCl_3_, 300.13 MHz and 75.47 MHz) and HMBC assignment for **1**.

Position	δ_C_, Type	δ_H_, (*J* in Hz)	COSY	HMBC
1	38.2, CH_2_	1.05, m	H-2	
2	23.3, CH_2_	1.65, m	H-1, 3	
		1.33, dd (12.9, 4.2)	H-1, 3	C-4
3	80.8, CH	4.48, dd (10.9, 4.6)	H-2	C-1, 4, 21, 22
4	37.8, C	-		
5	55.4, CH	0.91, dd (12.0, 2.2)	H-6	
6	18.7, CH_2_	1.62, m	H-5	
		1.14, m		
7	40.5, CH_2_	1.18, dd (12.5, 3.6)		
		1.88, m		
8	39.8, C	-		
9	59.8, CH	1.02, dd (12.3, 2.6)		
10	37.4, C	-		
11	23.6, CH_2_	1.70, m		
12	38.0, CH_2_	2.38, m		
		1.92, m		C-14, 25
13	147.7, C	-		
14	56.4, CH	1.59, m	H-15	
15	19.6, CH_2_	1.86, m	H-14, 16	C-13
16	37.2, CH_2_	2.08, m	H-15	
17	194.7, CO	-		
18	99.9, CH	5.45, s		C-16, 17, 19, 20
19	191.1, C	-		
20	24.9, CH_3_	2.05, s		C-18, 19
21	16.3, CH_3_	0.83, s		C-3, 4, 5, 22
22	28.0, CH_3_	0.86, s		C-3, 4, 5, 21
23	16.4, CH_3_	0.84, s		C-1, 5, 9, 10
24	15.3, CH_3_	0.69, s		C-7, 8, 9, 14
25a	106.4, CH_2_	4.84, brs		C-12, 14
b		4.50, brs		C-12, 13, 14
26	171.0, CO	-		
27	21.3, CH_3_	2.05, s		C-26
OH-19		15.47, s		

**Figure 3:**
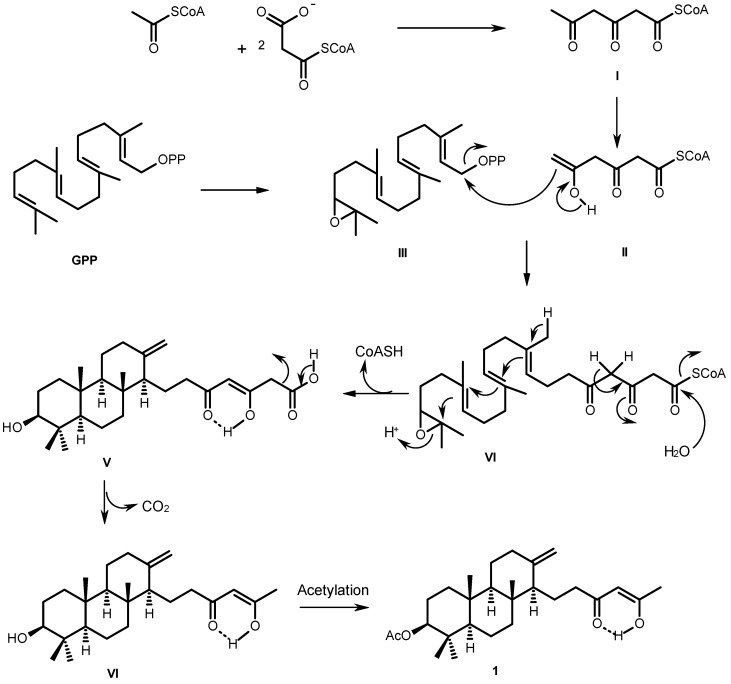
Proposed biogenesis of sartorenol (**1**).

The biosynthetic pathway of sartorenol (**1**) resembles those proposed for aszonapyrone A and sartorypyone A [6], which is hypothesized as originating from a reaction of the triketide derivative (**II**) with GPP oxide (**III**) to form the meroditerpene intermediate (**IV**). Cyclization, hydrolysis of the CoA ester and enolization of the side chain give the intermediate (**V**). Decarboxylation of the side chain and acetylation of the hydroxyl group of the perhydrophenanthrene moiety would finally lead to the formation of sartorenol (**1**) (Figure 3).

Compound **2** was isolated as white solid (mp, 182–183 °C), and its molecular formula C_23_H_23_N_3_O_2_ was established on the basis of the (+)-HRESIMS *m*/*z* 374.1876 [M + H]^+^ (calculated for C_23_H_24_N_3_O_2_, 374.1869), indicating fourteen degrees of unsaturation. The IR spectrum showed absorption bands for amine (3214 cm^−1^), amide carbonyls (1688, 1654 cm^−1^), aromatic (3057, 1579 cm^−1^) and olefin (1607, 1468 cm^−1^) groups. The ^13^C NMR, DEPTs and HSQC spectra (Table 2, Supplementary Figures S9 and S10) revealed the presence of two amide carbonyls (δ_C_172.0 and 168.9), six quaternary sp^2^ (δ_C_ 136.4, 136.3, 135.7, 127.9, 125.5, 108.0), ten methine sp^2^ (δ_C_ 133.1, 131.4, 127.3, 125.2, 121.7, 121.0, 119.9, 119.2, 118.4, 109.9), one methine sp^3^ (δ_C_ 52.4), two methylene sp^3^ (δ_C_ 44.2 and 22.4) and two methyl (δ_C_ 25.6 and 18.1) carbons. The coupling system of the aromatic protons, observed in the COSY spectrum (Table 2, Supplementary Figure S8), indicated the presence of two 1,2-disubstituted benzene rings. That one of the 1,2-disubstituted benzene rings was part of the 3,4-dihydro-1*H*-1,4-benzodiazepine-2,5-dione was supported by the HMBC cross peaks (Table 2, Supplementary Figure S11) of the singlet of the amine proton at δ_H_ 9.03 (NH-16) to C-11 (δ_C_ 52.4) and C-14 (δ_C_ 125.5), of H-21 (δ_H_ 7.91, dd, *J* = 8.0, 1.5 Hz) to C-13 (δ_C_ 168.9), as well as by the COSY cross peaks (Table 2, Supplementary Figure S8) observed between NH-12 (δ_H_ 7.03, brd, *J* = 5.5 Hz) and H-11 (δ_H_ 4.12, dt, *J* = 8.3, 5.5 Hz). That another 1,2-disubstituted benzene ring belonged to the indole moiety of the molecule was substantiated by the HMBC cross peaks (Table 2, Supplementary Figure S11) of H-2 (δ_H_ 7.15, s) to C-3 (δ_C_ 108.0), C-8 (δ_C_ 136.3) and C-9 (δ_C_ 127.9). The presence of the 3-methylbuten-2-yl moiety was corroborated by cross peaks of H-1′ (δ_H_ 4.63, d, *J* = 6.8 Hz) to H-2′ (δ_H_ 5.35, m), CH_3_-4′ (δ_H_ 1.74, s) and CH_3_-5′ (δ_H_ 1.80, s) protons in the COSY spectrum, as well as by the HMBC cross peaks of CH_3_-4′ (1.74, s) and CH_3_-5′ (1.80, s) protons to C-2′ (δ_C_ 119.9) and C-3′ (δ_C_ 136.4). Since the HMBC spectrum showed cross peaks of H-1′ to C-2 (δ_C_ 127.3) and C-8 (δ_C_ 136.3), the 3-methylbuten-2-yl moiety was linked to the indole nitrogen. That the indole moiety was linked to the 3,4-dihydro-1*H*-1,4-benzodiazepine-2,5-dione by a methylene bridge, through C-3 of the former and C-11 of the latter, was evidenced by the COSY correlations of H_2_-10 (δ_H_ 3.57, dd, *J* = 15.2, 5.5 Hz and 3.26, dd , *J* = 15.2, 8.3 Hz) to H-11 (δ_H_ 4.12, dt, *J* = 8.3, 5.5 Hz), as well as by the HMBC cross peaks of H_2_-10 to C-2, C-3 (δ_C_ 108.0) and C-17 (δ_C_ 172.0). 

A literature search revealed that the compound (3*S*)-3-[1-(3-methylbut-2-enyl)indol-3-yl]-3,4-dihydro-1*H*-1,4-benzodiazepine-2,5-dione (PubChem SID 185030170), whose flat structure is the same as that of compound **2**, was reported as a product of Angene Chemical (AGN-PC-069E9V) [11]. Although the absolute configuration of its C-11 is reported as *S*, there is neither ^1^H/^13^C NMR nor optical rotation data available for this compound in the PubChem Substance website. Since compound **2** did not provide suitable crystals for X-ray diffraction, it was not possible to determine the absolute configuration of C-11 with certainty. Thus, an attempt was made to combine the data from the NOESY spectrum, scalar coupling constants and molecular mechanics simulations.

**Table 2:** ^1^H and ^13^C NMR (DMSO, 300.13 MHz and 75.47 MHz) and HMBC assignment for **2**.

Position	δ_C_, Type	δ_H_, (*J* in Hz)	COSY	HMBC
2	127.3, CH	7.15, s		C-3, 8, 9
3	108.0, C	-		
4	118.4, CH	7.54, d (7.8)	H-5	C-3, 6, 8
5	119.2, CH	7.08, ddd (7.8, 7.8, 0.7)	H-4, 6	C-7, 9
6	121.7, CH	7.20, ddd (7.8, 7.8, 0.7)	H-5, 7	C-4, 8
7	109.9, CH	7.31, d (7.8)	H-6	C-5, 9
8	136.3, C	-		
9	127.9, C	-		
10	22.4, CH_2_	3.57, dd (15.2, 5.5)	H-11	C-2, 3, 17
		3.26, dd (15.2, 8.3)	H-11	C-2, 3, 17
11	52.4, CH	4.12, dt (8.3, 5.5)	H-10, NH-12	
13	168.9, CO	-		
14	125.5, C	-		
15	135.7, C	-		
17	172.0, CO			
18	121.0, CH	7.06, d (8.0)	H-19	C-14, 20
19	133.1, CH	7.50, ddd (8.0, 8.0, 1.5)	H-18, 20	C-15, 21
20	125.2, CH	7.24, dd (8.0, 8.0)	H-19, 21	C-14, 18
21	131.4, CH	7.91, dd (8.0, 1.5)	H-20	C-13, 19, 15
1′	44.2, CH_2_	4.63, d (6.8)	H-2′	C-2, 2′, 3′
2′	119.9, CH	5.35, m	H-1′, 4′, 5′	
3′	136.4, C	-		
4′	25.6, CH_3_	1.74, s	H-1′, 2′	C-2′, 3′, 5′
5′	18.1, CH_3_	1.80, s	H-1′, 2′	C-2′, 3′, 5′
NH-12		7.03, d (5.5)	H-11	
NH-16		9.03, s		C-11, 14

**Figure 4:**
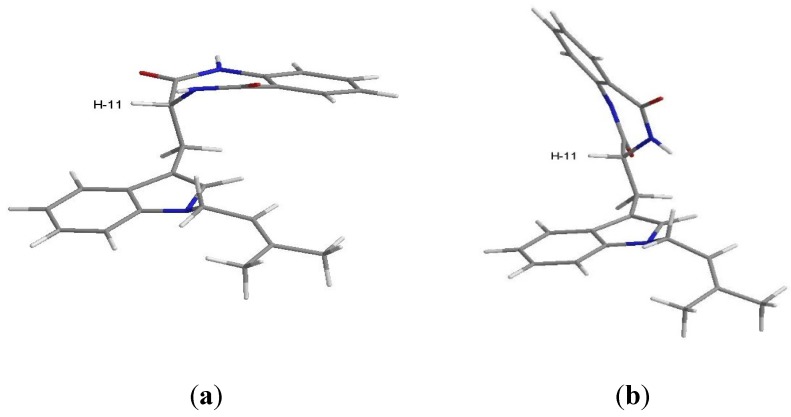
Conformations of C-11*R* stereoisomer of compound **2** obtained by simulation performed with ChemBio3D Ultra 14.0; (**a**) conformer with H-11 in equatorial position; (**b**) conformer with H-11 in axial position.

The NOESY spectrum (Supplementary Figure S12) exhibited correlations of H-11 to H-4, NH-12 and NH-16. A stochastic conformational search using MMFF force field models of the C-11 stereoisomers of compound **2**, performed with ChemBio3D Ultra 14.0 using the MMFF force field with application’s default parameters [12] showed a somewhat flat energy landscape concerning the spatial relative positions of the two cyclic regions of the molecule. The rotational freedom around the two carbon-carbon single bonds of C-10, on which compound **2** whole conformations hinge, precludes any clear differentiation between the two stereoisomers since both C-11*R* and C-11*S* stereoisomers yield lowest energy conformations that explain the observed NOESY cross-peaks as well as the ^1^H scalar coupling constants measured. Regardless of the stereoisomer, the gas-phase least energetic conformers of compound **2** show almost equal steric energy for the two major conformations (half-chair) of the amide ring. Figure 4 shows the C-11*R* stereoisomer as an example: (a) H-11 is in the equatorial and (b) H-11 is in the axial position. The major difference resides in the dihedral angle between H-11 and NH-12, which is approximately 0° for the equatorial and 110° for the axial position of H-11 relative to the ring. The observed scalar coupling of 5.5 Hz between the two protons may be interpreted as an average value between their extreme relative positions, suggesting that the two conformations exchange rapidly at room temperature. The observed NOESY correlation between H-11 and NH-16 does not allow us to positively decide for any of the two amide ring conformations since the distances between the two protons in the molecular mechanics models are very close, *i.e.*, 4.0 Å for the equatorial H-11, and 3.6 Å for the axial H-11. Therefore, the constant exchange between the two conformations of the amide ring is the most probable case.

However, the co-occurrence of compound **2** with aszonalenin and acetylaszonalenin in this extract suggested that they should be derived from the same biosynthetic pathways. Thus, it is probable that the absolute configuration of C-11 of compound **2** is the same as that of the corresponding carbons of aszonalenin and acetylaszonalenin, *i.e.*, 11*R*. Thus, compound **2** is a new natural product and we have named it takakiamide.

**Figure 5:**
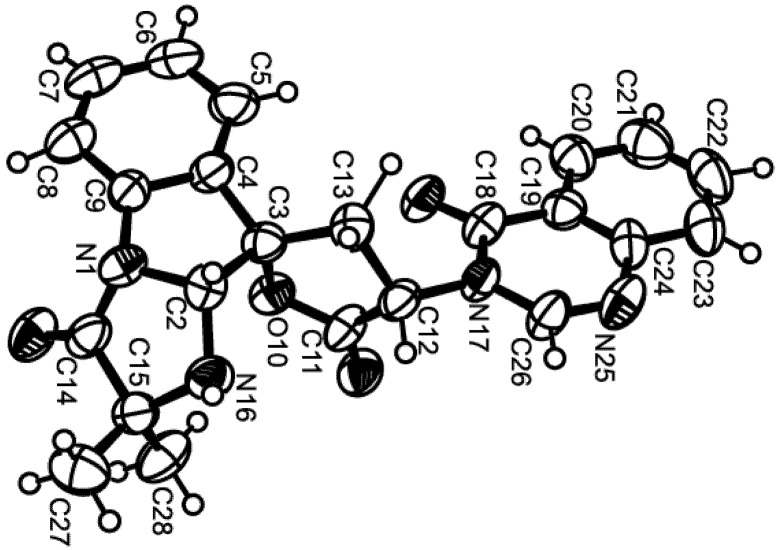
Ortep view of tryptoquivaline U (**3**).

Compound **3** was isolated as white crystals (mp, 208–209 °C), and its molecular formula C_23_H_21_N_4_O_4_ was established on the basis of the [M + H]^+^ peak at *m*/*z* 417.1563 (calculated 417.1563) in the (+)-HRESIMS. The ^1^H and ^13^C NMR spectra of compound **3** (Table 3, Supplementary Figures S13 and S15) resembled those of tryptoquivaline L [9]. The ^13^C NMR, DEPT and HSQC spectra (Table 3, Supplementary Figures S15 and S16) displayed signals of three carbonyls (δ_C_ 176.0, 170.7, 159.6), four quaternary sp^2^ (δ_C_ 147.5, 139.8, 132.0, 121.4), nine methine sp^2^ (δ_C_ 147.4, 135.0, 131.6, 127.6, 127.3, 126.1, 125.7, 125.7, 116.2), two quaternary sp^3^ (δ_C_ 84.7 and 64.6), two methine sp^3^ (δ_C_ 82.0 and 56.9), one methylene sp^3^ (δ_C_ 31.6) and two methyl (δ_C_ 26.9 and 26.5) carbons. The ^1^H NMR and COSY spectra (Table 3, Supplementary Figures S13 and S14) revealed the presence of two 1,2-disubstituted benzene rings of the *gem*-dimethyl imidazoindolone ring system and quinazolin-4(3*H*)-one moiety as well as the protons of the five-membered spirolactone ring, similar to those of tryptoquivaline L [9]. However, contrary to tryptoquivaline L, H-2 of compound **3** appeared as a doublet at δ_H_ 5.55 (*J* = 8.4 Hz) instead of a singlet at δ_H_ 5.25 [9]. Moreover, the COSY spectrum exhibited a correlation between H-2 signal and a doublet at δ_H_ 3.76 (*J* = 8.4 Hz). Consequently, this signal was attributed to NH-16. Interestingly, both CH_3_-27 (δ_C_ 26.5) and CH_3_-28 (δ_C_ 26.9) resonated at higher chemical shift values than their counterparts in tryptoquivaline L (δ_C_ 16.4 and 22.8) while C-15 exhibited lower chemical shift value (δ_C_ 64.6) than the corresponding carbon (δ_C_ 70.0) of tryptoquivaline L [9]. Thus, the only difference between the structure of compound **3** and that of tryptoquivaline L is the presence of a hydrogen atom on N-16 instead of a hydroxyl group. This was supported by the molecular formula of compound **3** (C_23_H_20_N_4_O_4_), which has one oxygen atom less than that of tryptoquivaline L. In order to verify if the stereochemistry of compound **3** is the same as that of tryptoquivaline L, X-ray analysis of compound **3** was performed. The ORTEP diagram of compound **3** (Figure 5) showed unambiguously that the absolute configurations of C-2, C-3 and C-12 are *S*, *S* and *R*, the same as that of the corresponding carbons of tryptoquivaline L. Since compound **3** is a new analog of tryptoquivalines, and in accordance with the names given to the tryptoquivaline series, we have named compound **3** tryptoquivaline U.

**Table 3:** ^1^H and ^13^C NMR (DMSO, 300.13 MHz and 75.47 MHz) and HMBC assignment for tryptoquivaline U (**3**).

Position	δ_C_, Type	δ_H_, (*J* in Hz)	COSY	HMBC
2	82.0, CH	5.55, d (8.4)	NH-16	C-13, 14
3	84.7, C	-		
4	132.0, C	-		
5	125.7, CH	7.71, d (7.3)	H-6	C-7, 9
6	125.7, CH	7.38, ddd (7.5, 7.5, 1.2)	H-5, 7	C-4, 8
7	131.6, CH	7.57, ddd (8.1, 7.7, 1.2)	H-6, 8	C-5, 9
8	116.2, CH	7.49, d (7.2)	H-7	C-4, 6
9	139.8, C	-		
11	170.7, CO	-		
12	56.9, CH	5.58, dd (10.8, 9.1	H-13	C-3, 11, 13, 18, 26
13	31.6, CH_2_	2.86, dd (12.9, 9.1)	H-12	C-2, 4, 11, 12
		3.45, dd (12.9, 11.2)	H-12	C-2, 3, 4, 12
14	176.0, CO	-		
15	64.6, C	-		
16	-	3.76, d (8.4)	H-2	C-2, 3, 14, 15, 26, 27
18	159.6, CO	-		
19	121.4, C	-		
20	126.1, CH	8.23, dd (8.0, 1.2)	H-21	C-18, 22, 24
21	127.6, CH	7.63, ddd (7.6, 7.6, 1.0)	H-20, 22	C-19, 23
22	135.0, CH	7.92, ddd (8.2, 8.2, 1.5)	H-21, 23	C-20, 24
23	127.3, CH	7.76, d (7.7)	H-22	C-19, 21
24	147.5, C	-		
26	147.4, CH	8.49, s		C-12, 18, 24
27	26.5, CH_3_	1.45, s		C-14, 15, 28
28	26.9, CH_3_	1.24, s		C-14, 15, 27

Since we have previously found that the meroditerpenes aszonapyrone A and sartorypyrone A, isolated from the culture of *N. fischeri* KUFC 6344, exhibited potent antibacterial activity as well as synergism with antibiotics against the Gram-positive multidrug-resistant strains [7], we also evaluated sartorenol (**1**), takakiamide (**2**) and tryptoquivaline U (**3**) for their antibacterial activity against four reference strains (*Staphylococcus aureus*, *Bacillus subtillis*, *Escherichia coli* and *Pseudomonas aeruginosa*), as well as the environmental multidrug-resistant isolates, according to the previously described method [7]. The results showed that none of the tested compounds exhibited relevant antibacterial activity, *i.e.*, their MIC values are higher than 256 mg/mL. These compounds were also tested for their capacity to inhibit a quorum sensing by the screening protocol based on the pigment production by *Chromobacterium violaceum* ATCC 31532 [13] and none of them showed a quorum sensing inhibitory activity.

## 3. Experimental Section

### 3.1. General Procedure

Melting points were determined on a Bock monoscope and are uncorrected. Optical rotations were measured on an ADP410 Polarimeter (Bellingham + Stanley Ltd., Tunbridge Wells, Kent, UK). Infrared spectra were recorded in a KBr microplate in a FTIR spectrometer Nicolet iS10 from Thermo Scientific (Waltham, MA, USA) with Smart OMNI-Transmission accessory (Software 188 OMNIC 8.3). UV spectra were taken in CHCl_3_ and were recorded on a Varian CARY 100 spectrophotometer. ^1^H and ^13^C-NMR spectra were recorded at ambient temperature on a Bruker AMC instrument (Bruker Biosciences Corporation, Billerica, MA, USA) operating at 300.13 and 75.4 MHz, respectively. High-resolution mass spectra were measured with a Waters Xevo QToF mass spectrometer (Waters Corporations, Milford, MA, USA) coupled to a Waters Aquity UPLC system. A Merck (Darmstadt, Germany) silica gel GF254 was used for preparative TLC, and a Merck Si gel 60 (0.2–0.5 mm) was used for analytical chromatography.

### 3.2. Extraction and Isolation

The strain KUFC 7898 was isolated from the alga *Amphiroa* sp., which was collected from Samaesarn Island in the Gulf of Thailand, Chonburi Province, in September 2011. The alga was washed with 0.06% sodium hypochlorite solution for 1 min, followed by sterilized seawater three times. The alga was dried on sterile filter paper, cut into small pieces (5 × 5 mm) and placed on a malt extract agar (MEA) medium containing 70% seawater and incubated at 28 °C for 5–7 days. The fungus was identified as *Neosartorya takakii*, by Leka Manoch (Department of Plant Pathology, Faculty of Agriculture, Kasetsart University, Bangkok, Thailand), based on morphological characteristics such as colony growth rate and growth pattern on standard media, namely Czapek’s agar (CZA), Czapek yeast autolysate agar (CYA) and malt extract agar (MEA). Microscopic characteristics including size, shape, ornamentation of ascospores and *Aspergillus takakii* anamorph were examined under light and scanning electron microscopes [14]. This identification was supported by sequence analysis of the β-tubulin, calmodulin and actin genes as described in the previous report [15]. *Neosartorya takakii* was also confirmed by analysis sequence of the internal transcribed spacer (ITS) gene. Briefly, 2–15 mg of mycelia was ground in liquid nitrogen. DNA was extracted using the DNeasy™ Plant Mini Kit (QIAGEN, Hilden, Germany) according to the manufacturer’s instructions. The entire nuclear ITS regions were amplified with the primers: ITS1F-5′ [16] and ITS4-3′ [17]. PCR reactions were conducted on Thermal Cycler and the amplification process consisted of initial denaturation at 95 °C for 5 min, 34 cycles at 95 °C for 1 min (denaturation), at 55 °C for 1 min (annealing) and at 72 °C for 1.5 min (extension), followed by final extension at 72 °C for 10 min. PCR products were cleaned using QIAquick PCR Purification Kit (QIAGEN, Hilden, Germany), then examined by Agarose gel electrophoresis (1% agarose with 1× TBE buffer) and visualized under UV light after staining with ethidium bromide. DNA sequencing analyses were carried out by Macrogen Inc. (Seoul, South Korea). The sequences were compared using the NCBI nucleotide BLAST program (http://www.ncbi.nlm.nih.gov/BLAST) for identification [18]. The pure cultures were deposited as KUFC 7898 at Kasetsart University Fungal Collection, Department of Plant Pathology, Faculty of Agriculture, Kasetsart University, Bangkok, Thailand, and also as MMERU 03 at Microbes Marine Environment Research Unit, Division of Environmental Science, Faculty of Science, Ramkhamhaeng University, Bangkok, Thailand.

The fungus was cultured for one week at 28 °C in 10 Petri dishes (i.d. 90 mm) containing 25 mL of MEA with 70% seawater per dish. Fifty 1000 mL Erlenmeyer flasks, each containing rice (200 g), water (30 mL), and seawater (70 mL), were autoclaved, inoculated with five mycelia plugs of *N. takakii* and incubated at 28 °C for 30 days, after which the moldy rice was macerated in ethyl acetate (15 L total) for 10 days and then filtered. The two layers were separated using a separating funnel and the ethyl acetate solution was concentrated under reduced pressure to yield 83.5 g of crude ethyl acetate extract which was dissolved in 500 mL of CHCl_3_ and then washed with 5% NaHCO_3_ aqueous solution (2 × 300 mL) and H_2_O (3 × 300 mL). The organic layers were combined and dried with anhydrous Na_2_SO_4_, filtered and evaporated under reduced pressure to give 53.8 g of the crude chloroform extract, which was applied on a column of silica gel (420 g), and eluted with mixtures of petrol–CHCl_3_ and CHCl_3_–Me_2_CO, 250 mL fractions were collected as follows: Frs 1–40 (petrol–CHCl_3_, 1:1), 41–82 (petrol–CHCl_3_, 3:7), 83–197 (petrol–CHCl_3_, 1:9), 198–321 (CHCl_3_–Me_2_CO, 9:1), and 322–460 (CHCl_3_-Me_2_CO, 7:3). Frs 198–203 were combined (1.57 g) and applied over a column chromatography of silica gel (35 g) and eluted with mixtures of petrol–CHCl_3_, CHCl_3_–Me_2_CO and Me_2_CO, 200 mL sub-fractions were collected as follows; sfrs 1–80 (petrol-CHCl_3_, 1:1), 81–110 (petrol–CHCl_3_, 3:7), 111–138 (petrol–CHCl_3_, 1:9), 139–150 (CHCl_3_–Me_2_CO, 9:1), and 151–154 (Me_2_CO). Sfrs 27–33 were combined (80.3 mg) and recrystallized in MeOH to give 26.7 mg of sartorenol (**1**). Sfrs 34–70 were combined (498 mg) and purified by TLC (silica gel G_254_, CHCl_3_–Me_2_CO–HCO_2_H, 9.5:0.5:0.1) to give an additional 18.2 mg of sartorenol (**1**). Sfrs 71–90 were combined (179.0 mg) and purified by TLC (silica gel G_254_, CHCl_3_–Me_2_CO–HCO_2_H, 9.5:0.5:0.1) to give chevalone B (33.6 mg) [7]. Sfrs 91–112 were combined (78.4 mg) and purified by TLC (silica gel G_254_, CHCl_3_–Me_2_CO–HCO_2_H, 9.5:0.5:0.1) to yield additional 2.7 mg of chevalone B. Frs 204–209 were combined (2.08 g) was recrystallized in MeOH to give aszonalenin (586.0 mg) [6], and the mother liquor was combined with frs 210–212 (1.53 g) and applied over a column chromatography of silica gel (35 g) and eluted with mixtures of petrol–CHCl_3_, CHCl_3_–Me_2_CO and Me_2_CO, wherein 200 mL sub-fractions were collected as follows: sfrs 1–25 (petrol–CHCl_3_, 1:1), 26–120 (petrol–CHCl_3_, 3:7). Sfrs 69–105 were combined (150.2 mg) and purified by TLC (silica gel G_254_, CHCl_3_–Me_2_CO–HCO_2_H, 9.5:0.5:0.1) to give 6-hydroxymellein (5 mg) [10]. Frs 213–224 were combined (626 mg) and crystallized in MeOH to give aszonapyraone A (230 mg) [6]. Frs 262–267 were combined (573.4 mg) and purified by TLC (silica gel G_254_, CHCl_3_–Me_2_CO–HCO_2_H, 8:2:0.1) to give 20.5 mg of takakiamide (**2**) and 91.3 mg of acetylaszonalenin [6]. Frs 268–283 were combined (1.03 g) and recrystallized in MeOH to give acetylaszonalenin (115.1 mg). Frs 325–334 were combined (2.95 g) and recrystallized in MeOH to give tryptoquivaline L (0.98 g) [9]. Frs 335–342 were combined (6.06 g) and recrystallized in MeOH to give tryptoquivaline H (259.5 mg) [9]. Frs 343–348 were combined (281 mg) and crystallized in MeOH to give 3′-(4-oxoquinazolin-3-yl) spiro[1*H*-indole-3,5′-oxolane]-2,2′-dione (24.9 mg) [9]. Frs 356–390 were combined (1.15 g) and purified by TLC (silica gel G_254_, CHCl_3_–Me_2_CO–HCO_2_H, 7:3:0.1) to give 16.5 mg of tryptoquivaline U (**3**) and 3.9 mg tryptoquivaline F [9]. Frs 391–400 were combined (125.3 mg) and recrystallized in MeOH to give 8.6 mg of tryptoquivaline F [9]. 

#### 3.2.1. Satorenol (**1**)

White crystal, Mp 122–123 °C (petrol-CHCl_3_); [α]_D_^20^ −18 (*c* 0.02, CHCl_3_); λ_max_ (log ε) 228 (4.41), 275 (3.99); IR (KBr) ν_max_ 3393, 2932, 2850, 1728, 1645, 1558, 1540, 1418, 1251 cm^−1^; ^1^H and ^13^C NMR (see Table 1); HRESIMS *m*/*z* 431.3175 (M + H)^+^ (calculated for C_27_H_43_O_4_, 431.3161).

#### 3.2.2. Takakiamide (**2**)

White solid, Mp 182–183 °C (petrol/CHCl_3_); [α]_D_^20^ −213 (*c* 0.06, CHCl_3_); IR (KBr) ν_max_ 3214, 3057, 2924, 2851, 1688, 1654, 1607, 1579, 1481, 1468, 1334, 1255 cm^−1^; ^1^H and ^13^C NMR (see Table 2); HRESIMS *m*/*z* 374.1876 (M + H)^+^ (calculated for C_23_H_24_N_3_O_2_, 374.1869).

#### 3.2.3. Tryptoquivaline U (**3**)

White crystals, Mp 208–209 °C (petrol/CHCl_3_); [α]_D_^20^ −196 (*c* 0.01, CHCl_3_); IR (KBr) ν_max_ 3363, 2924, 2852, 1775, 1710, 1662, 1607, 1473, 1384, 1260, 1199 cm^−1^; ^1^H and ^13^C NMR (see Table 3); HRESIMS 417.1563 (M + H)^+^ (calculated for C_23_H_21_N_4_O_4_, 417.1563).

### 3.3. X-Ray Crystal Structure of Sartorenol (**1**)

Crystals were orthorhombic, space group P*2*_1_*2*_1_*2*_1_, cell volume 2449.73(12) Å^3^ and unit cell dimensions *a* = 5.99830(16) Å, *b* =13.1349(3) Å and *c* = 31.0931(11) Å (uncertainties in parentheses). There are four molecules per unit cell with calculated density of 1.170 g/cm^−3^. Diffraction data were collected at 110 K with a Gemini PX Ultra equipped with CuK_α_ radiation (λ = 1.54184 Å). The structure was solved by direct methods using SHELXS-97 and refined with SHELXL-97 [19]. Carbon and oxygen were refined anisotropically. Hydrogen atoms bound to the carbon atom C20 were placed at their idealized positions using appropriate *HFIX* instructions in SHELXL, and included in subsequent refinement cycles. All other hydrogen atoms were directly found from difference Fourier maps and were refined freely with isotropic displacement parameters. The refinement converged to *R* (all data) = 9.51% and *wR*_2_ (all data) = 17.76%. Full details of the data collection and refinement and tables of atomic coordinates, bond lengths and angles, and torsion angles have been deposited with the Cambridge Crystallographic Data Centre (CCDC 1060934).

### 3.4. X-Ray Crystal Structure of Tryptoquivaline U (**3**)

Crystals were triclinic, space group P1, cell volume 501.08(12) Å^3^ and unit cell dimensions *a* = 5.3913(7) Å, *b* =9.8891(15) Å and *c* = 9.9063(13) Å and angles α = 84.939(11)°, β = 75.732(11)° and γ = 78.452(12)° (uncertainties in parentheses). There is one molecule per unit cell with calculated density of 1.380 g/cm^−3^. Diffraction data were collected at 293 K with a Gemini PX Ultra equipped with CuK_α_ radiation (λ = 1.54184 Å). The structure was solved by direct methods using SHELXS-97 and refined with SHELXL-97 [16]. Carbon and oxygen were refined anisotropically. Hydrogen atoms bound to carbon atoms C-5, C-6, C-20 and C-23 were placed at their idealized positions using appropriate *HFIX* instructions in SHELXL, and included in subsequent refinement cycles. All other hydrogen atoms were directly found from difference Fourier maps and were refined freely with isotropic displacement parameters. The refinement converged to *R* (all data) = 10.88% and *wR*_2_ (all data) = 30.04%. The absolute structure was established with confidence (flack *x* parameter 0.03(11)). Full details of the data collection and refinement and tables of atomic coordinates, bond lengths and angles, and torsion angles have been deposited with the Cambridge Crystallographic Data Centre (CCDC 1060935).

## 4. Conclusions

*N. takakii* has been previously isolated from a soil sample; however, this is the first report of the secondary metabolites from a marine-derived strain of this species. Besides the indole alkaloids aszonalenin; acetylaszonalenin; and tryptoquivalines L, H, and F, and the meroditerpene aszonapyrone A, which are common among the members of this genus, a new tryptoquivaline analog (trytoquivaline U), a new meroditerpene with an uncommon side chain containing an enol function (sartorenol), a prenylated indole alkaloid (takakiamide) and the previously reported isocoumarin derivative (6-hydroxymellein) were also produced by the culture of the marine-derived *N. takakii* KUFC 7898. Although sartorenol, takakiamide and tryptoquivaline U did not exhibit any antibacterial activity against the Gram-positive (*Staphylococcus aureus* ATCC 25923 and *Bacillus subtilis* ATCC 6633) and Gram-negative (*Escherichia coli* ATCC 25922 and *Pseudomonas aeruginosa* ATCC 27853) bacteria as well as methicillin-resistant *S. aureus* (MRSA) and vancomycin-resistant Enterococci (VRE) from the environment in our assay protocol, it does not mean that these new metabolites do not have other interesting biological activities. Thus, these new metabolites should be explored in other bioassay protocols so that their potential can be further discovered.

## Supplementary Files

Supplementary File 1
