# Supplementary material for: A New Meroditerpene and a New Tryptoquivaline Analog from the Algicolous Fungus Neosartorya takakii KUFC 7898
Source: Mar Drugs. 2015 Jun 15;13(6):3776–90. doi: 10.3390/md13063776 (PMC4483656; doi:10.3390/md13063776)
Supplement: Supplementary File 1 [file marinedrugs-13-03776-s001.pdf]

Supplementary Information

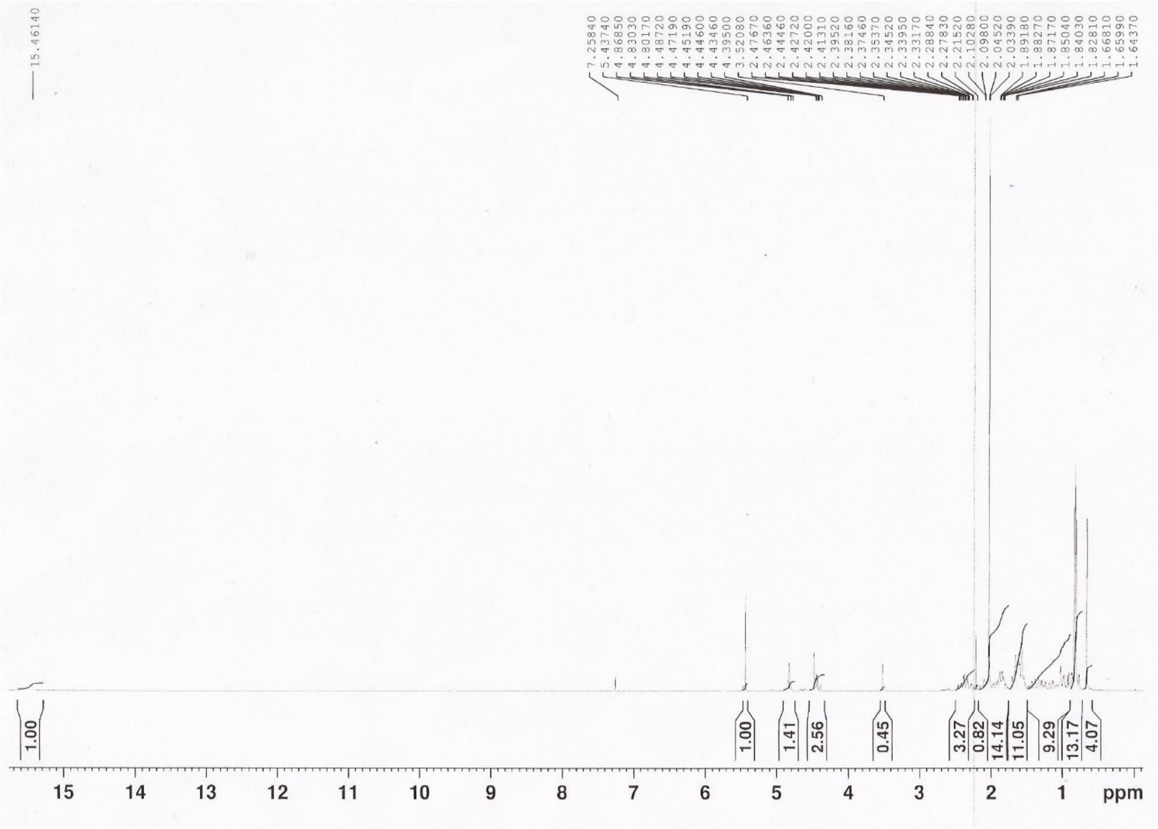

Figure S1. ^{1}H NMR spectrum of compound **1** ($CDCl_3$, 300.13 MHz).

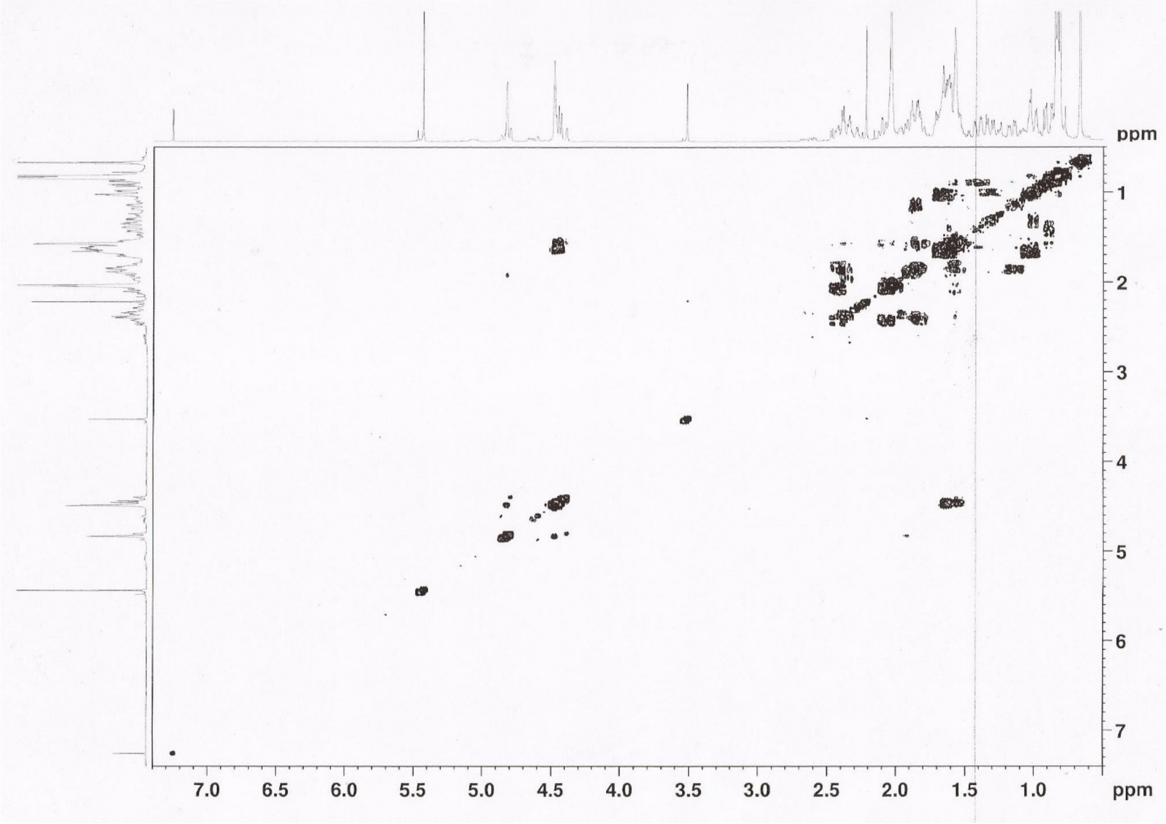

Figure S2. COSY spectrum of compound **1** ($CDCl_3$, 300.13 MHz).

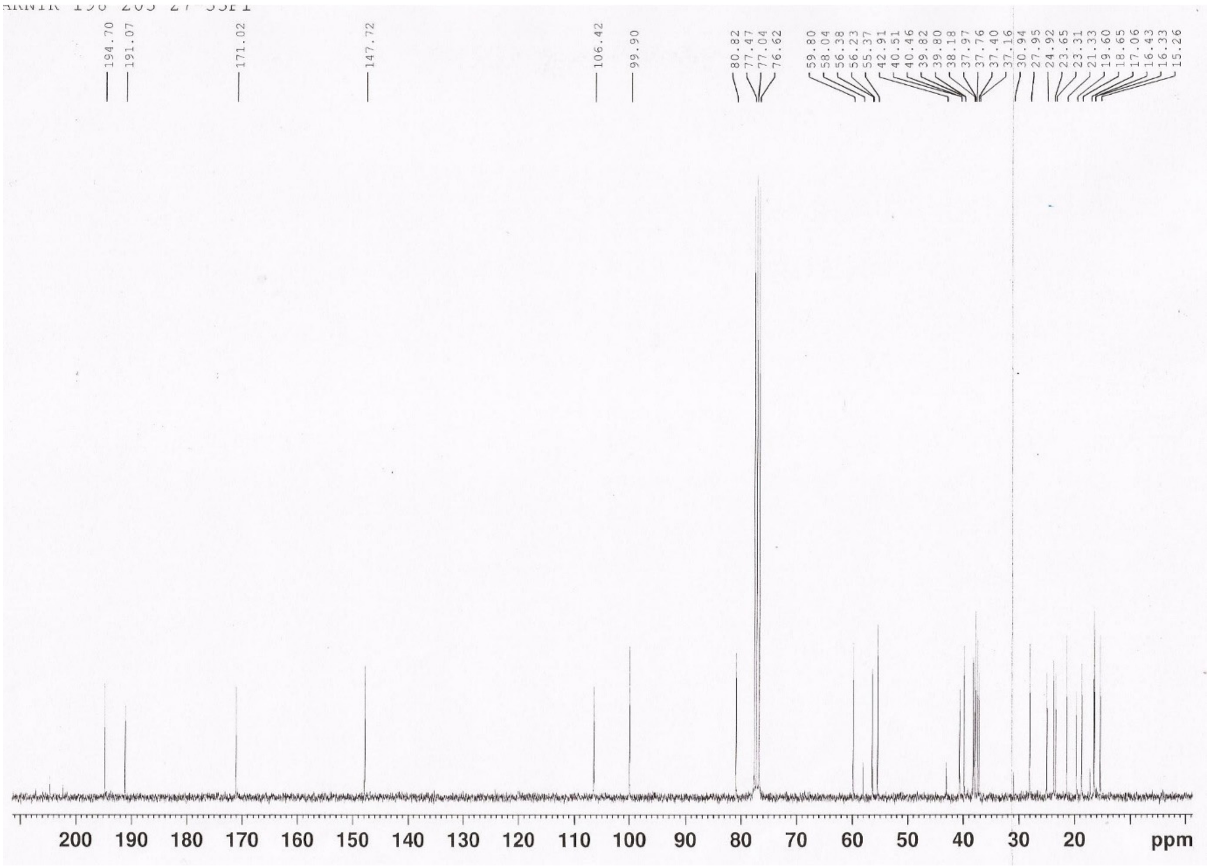

Figure S3. ^{13}C NMR spectrum of compound **1** ($CDCl_3$, 75.4 MHz).

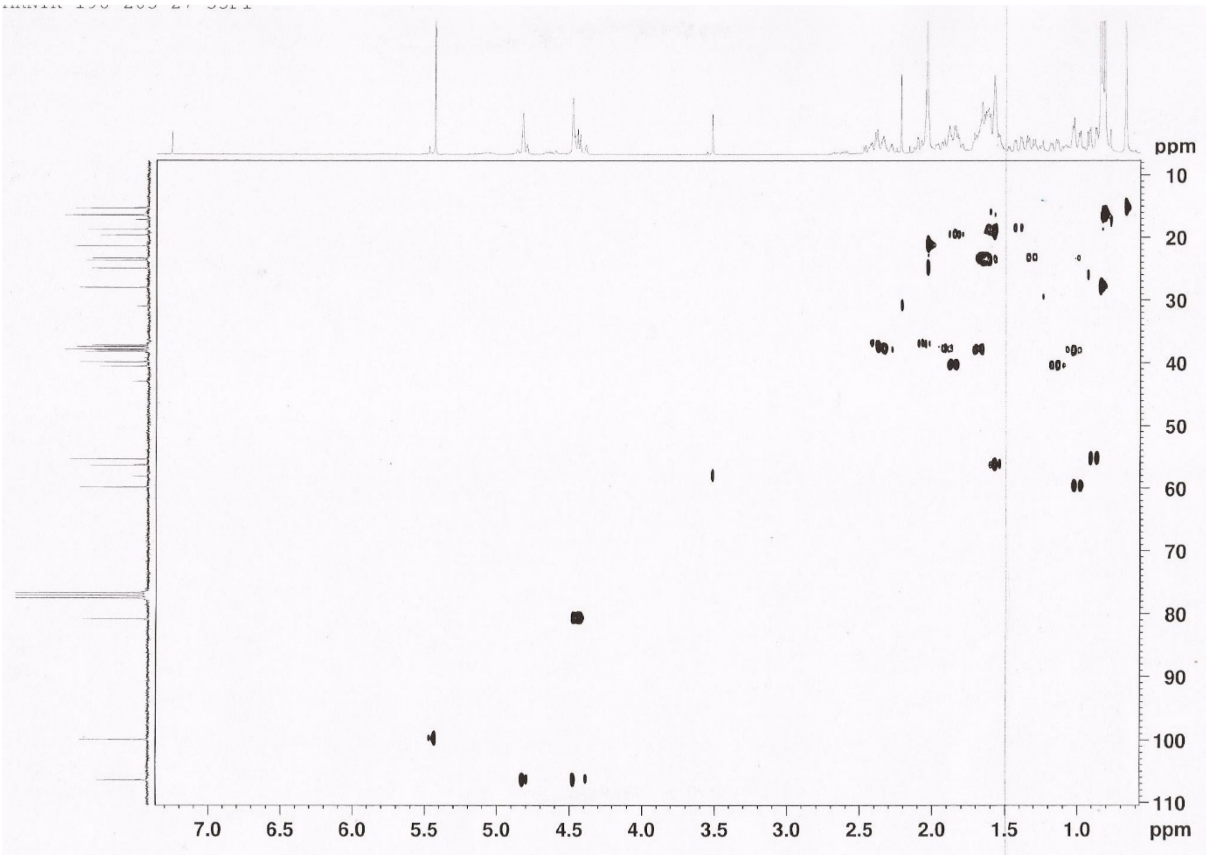

Figure S4. HSQC spectrum of compound **1** ($CDCl_3$, 300.13 MHz).

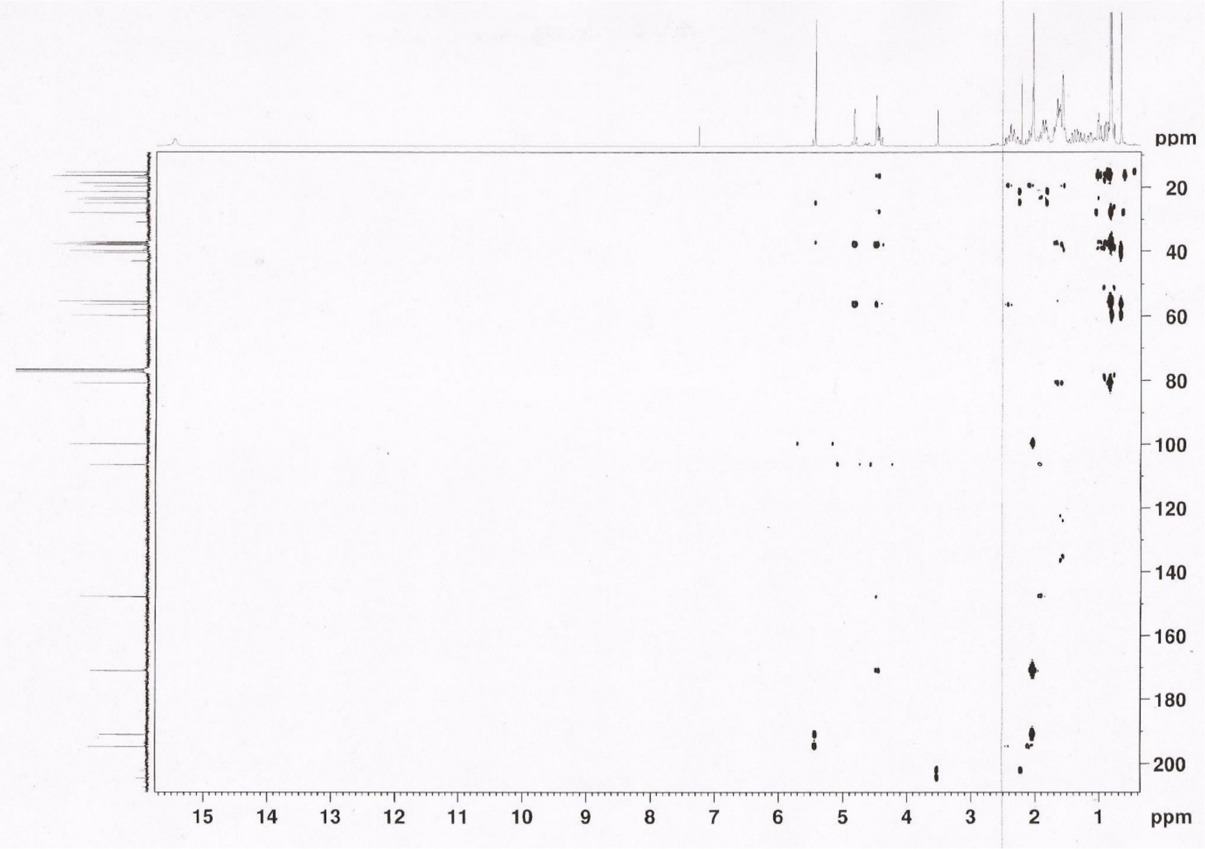

Figure S5. HMBC spectrum of compound **1** ($CDCl_3$, 300.13 MHz).

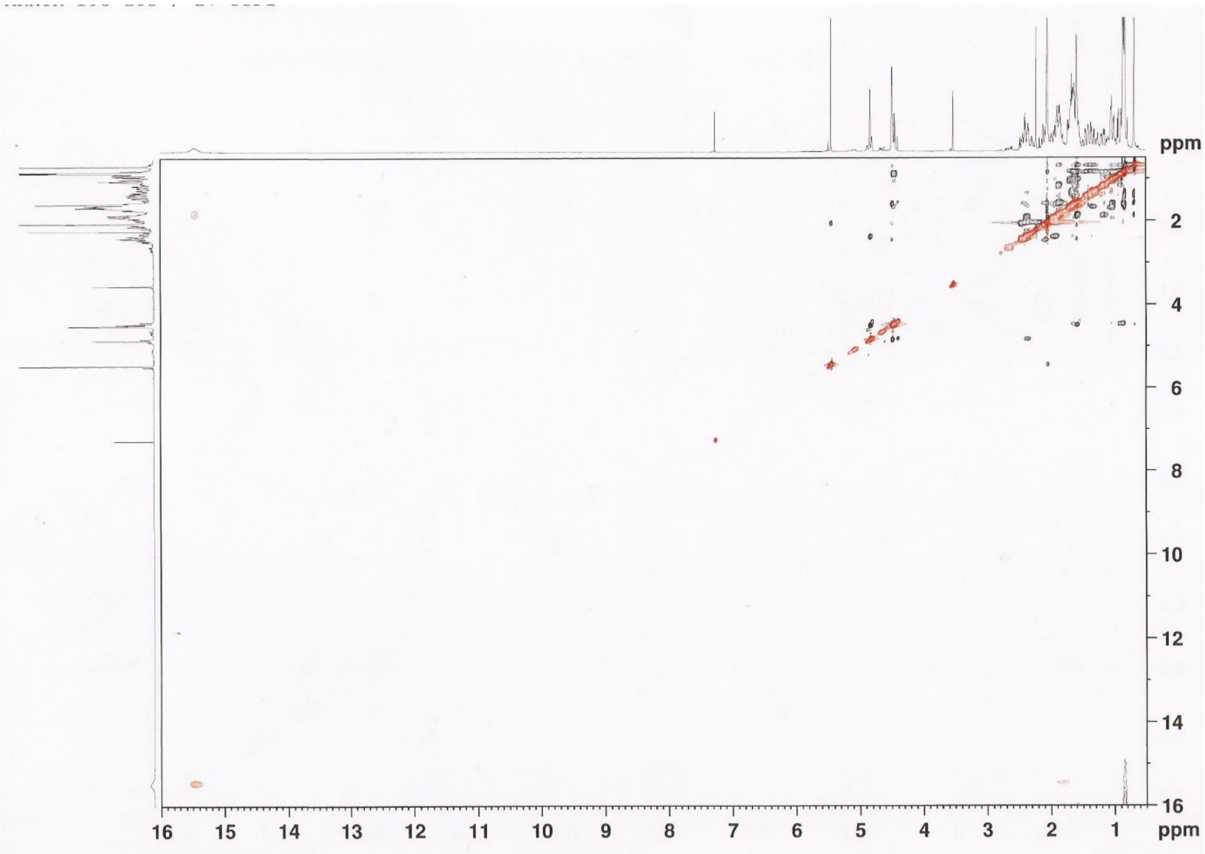

Figure S6. NOESY spectrum of compound **1** ($CDCl_3$, 300.13 MHz).

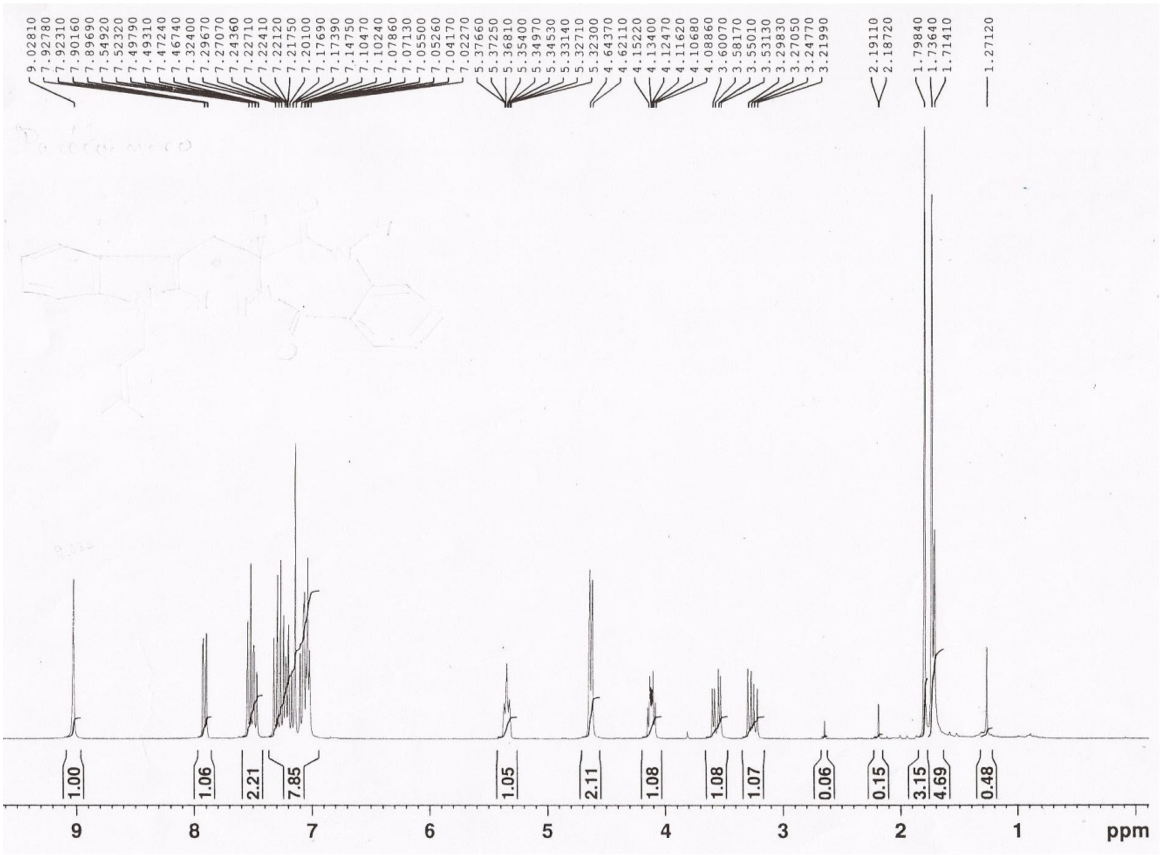

Figure S7. ^{1}H NMR spectrum of compound **2** ($CDCl_3$, 300.13 MHz).

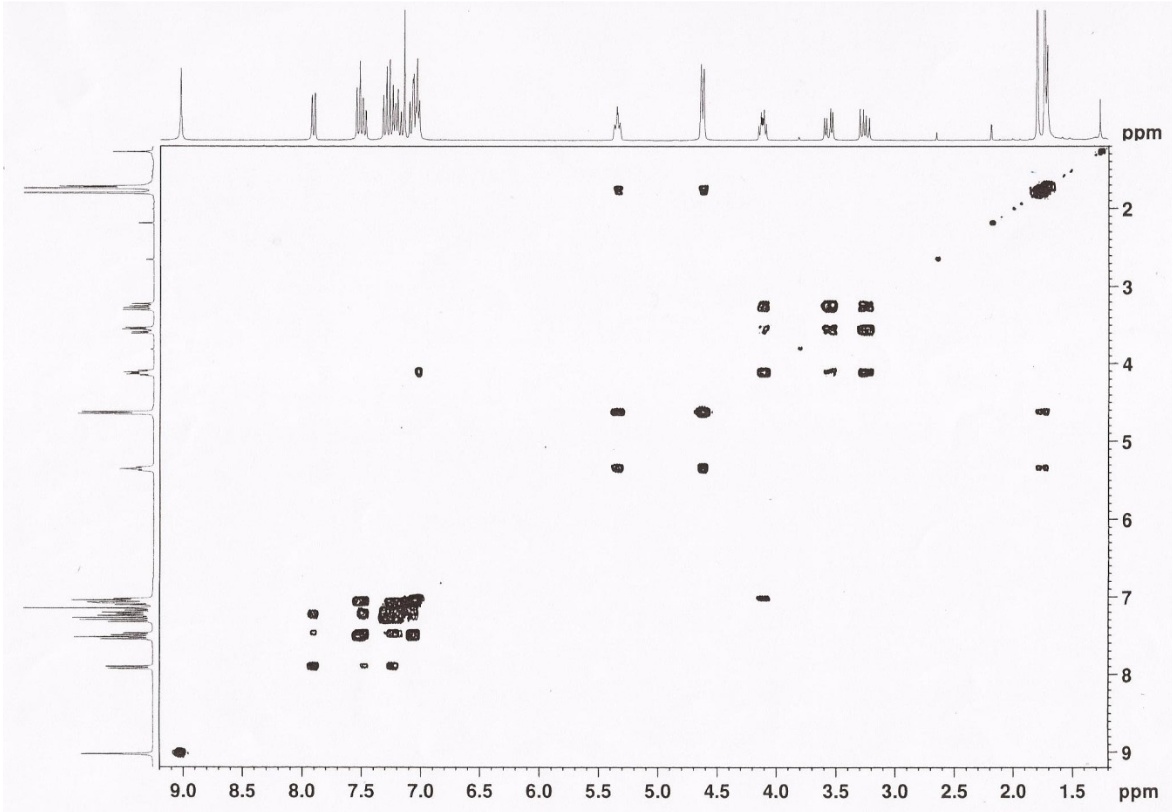

Figure S8. COSY spectrum of compound **2** ($CDCl_3$, 300.13 MHz).

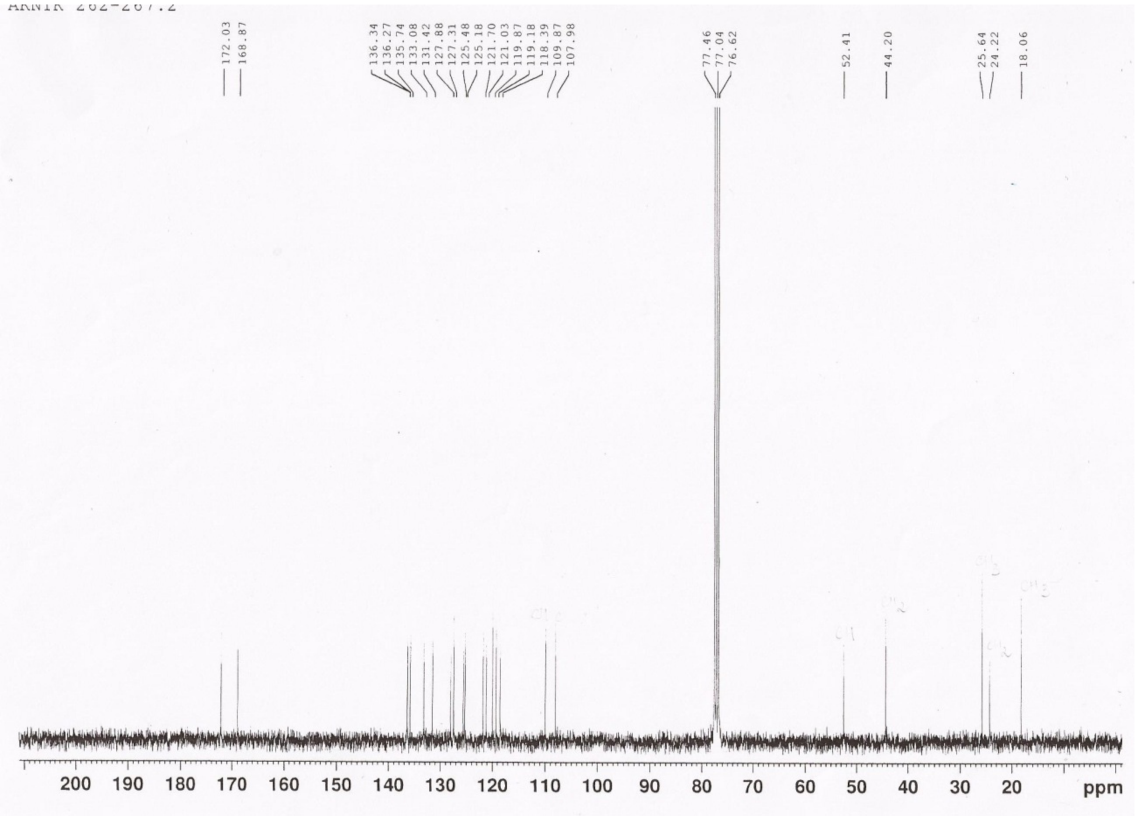

Figure S9. ^{13}C NMR spectrum of compound **2** ($CDCl_3$, 75.4 MHz).

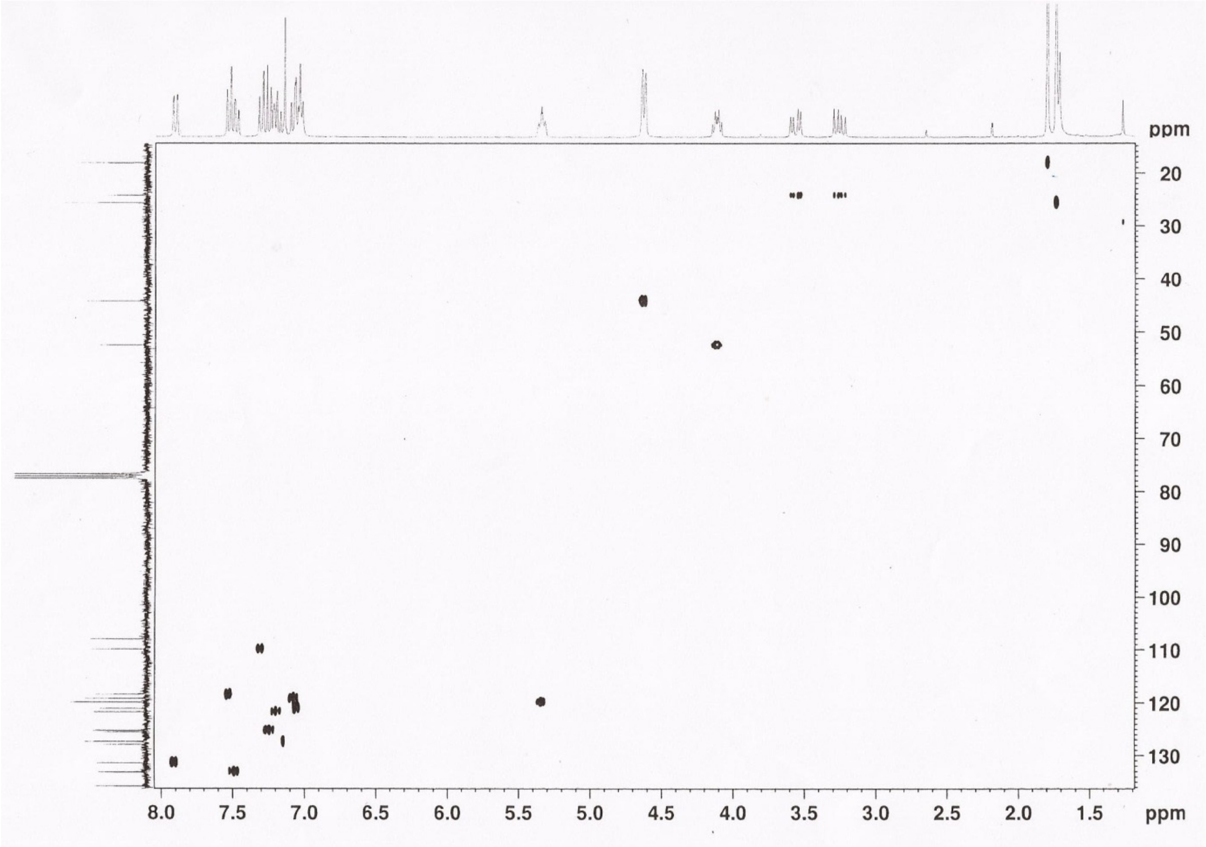

Figure S10. HSQC spectrum of compound **2** ($CDCl_3$, 300.13 MHz).

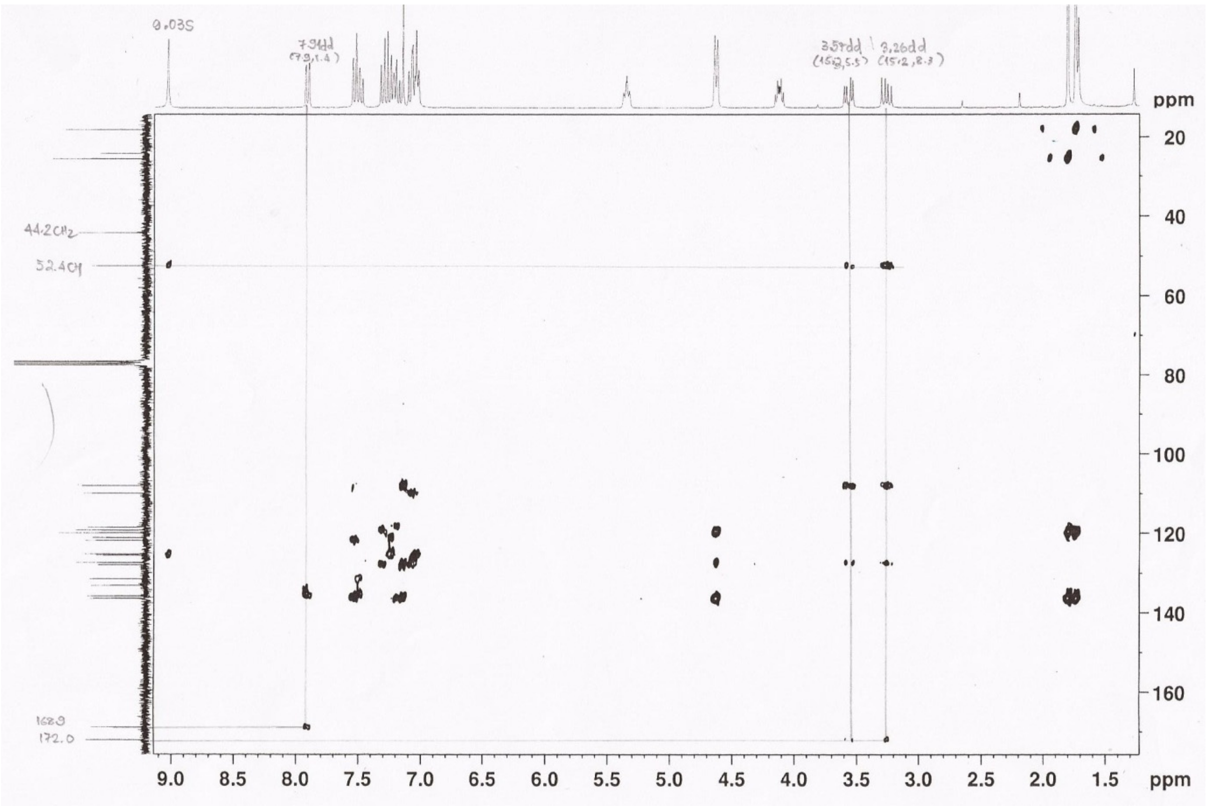

Figure S11. HMBC spectrum of compound **2** ($CDCl_3$, 300.13 MHz).

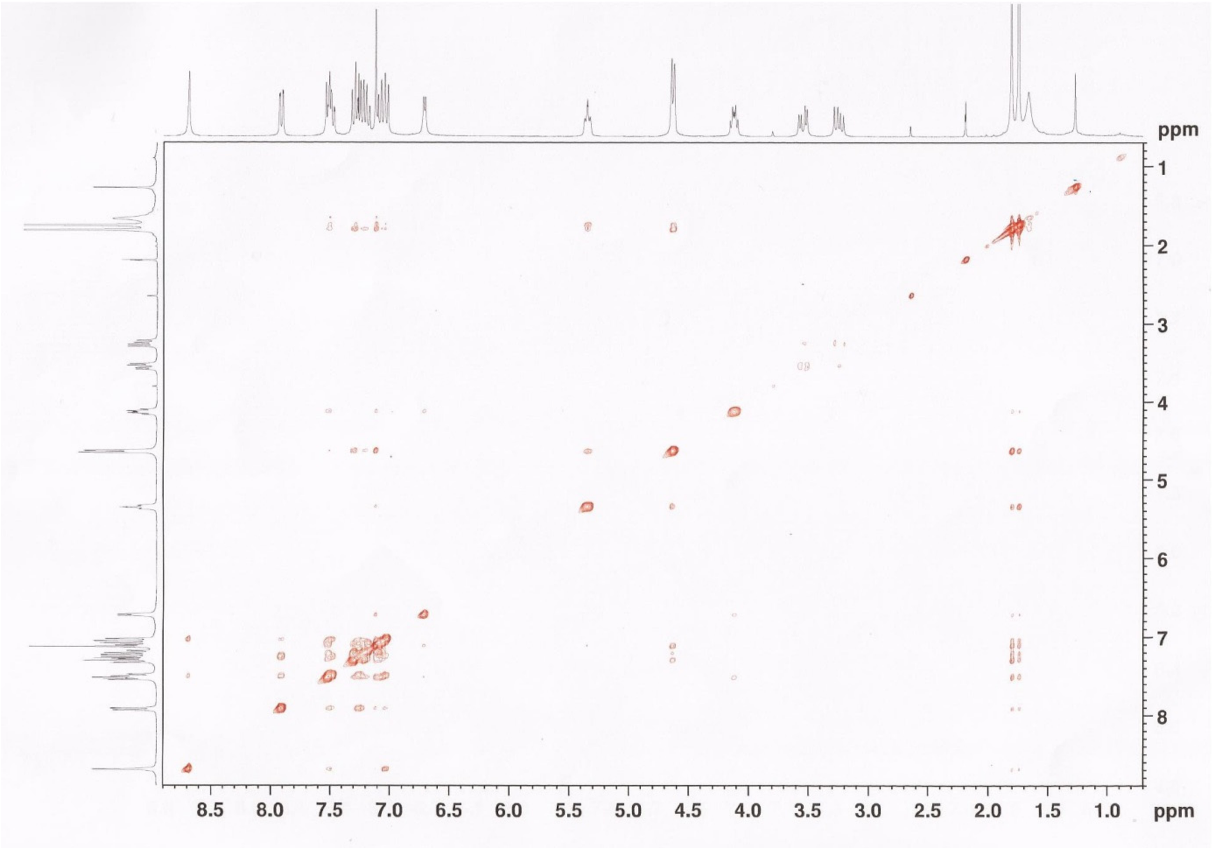

Figure S12. NOESY spectrum of compound **2** ($CDCl_3$, 300.13 MHz).

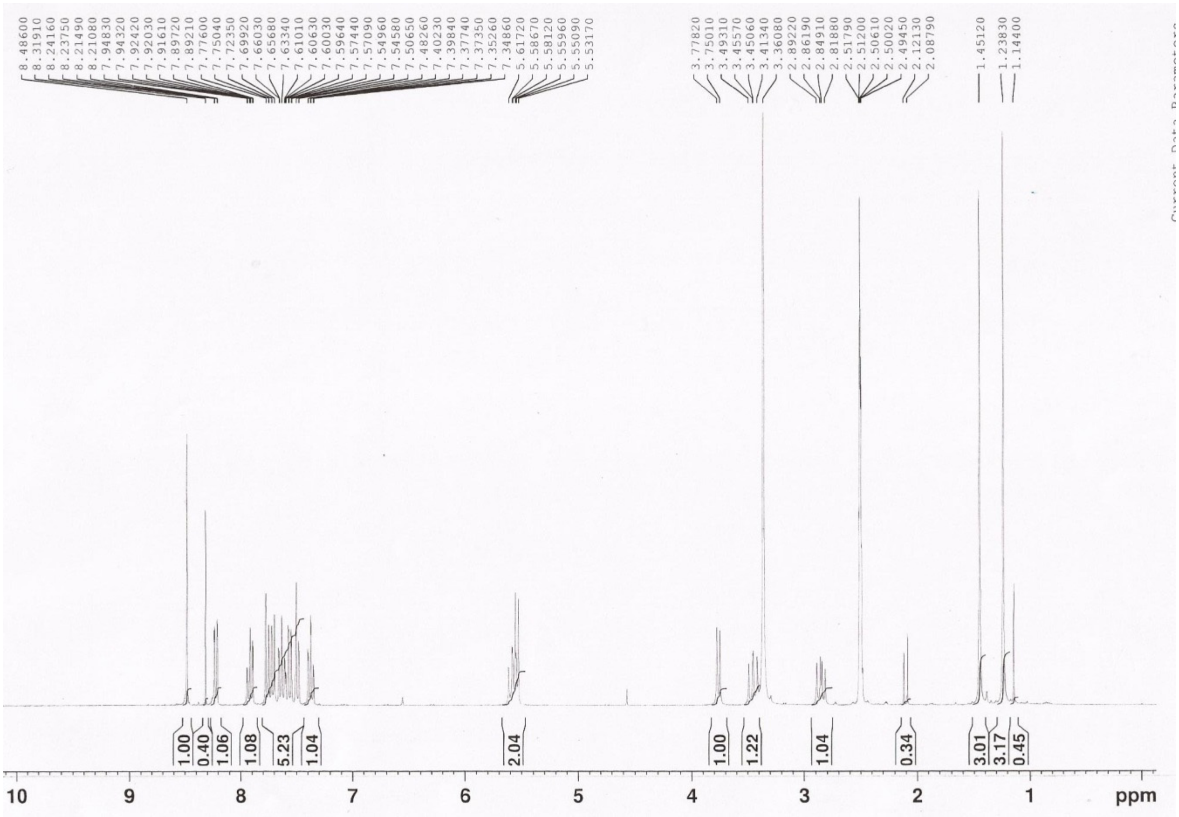

Figure S13. ^{1}H NMR spectrum of compound **3** (DMSO, 300.13 MHz).

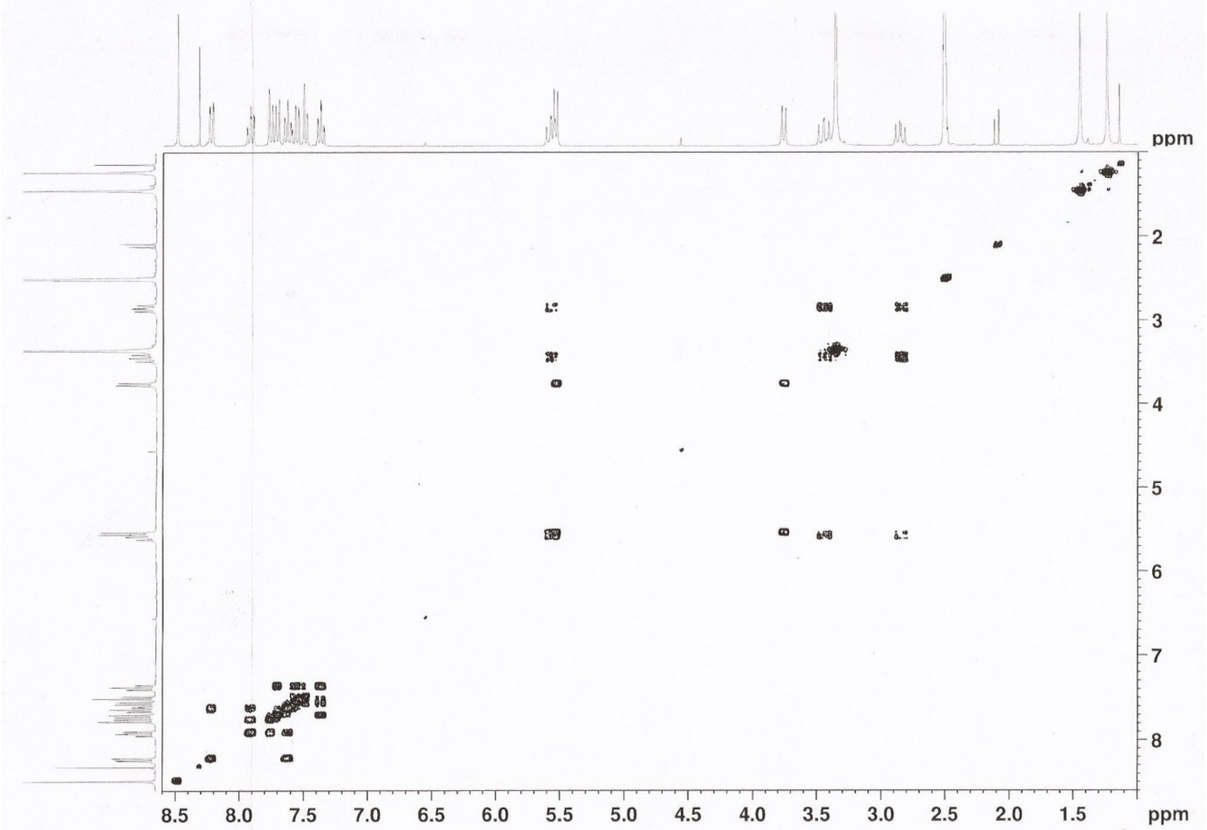

Figure S14. COSY spectrum of compound **3** (DMSO, 300.13 MHz).

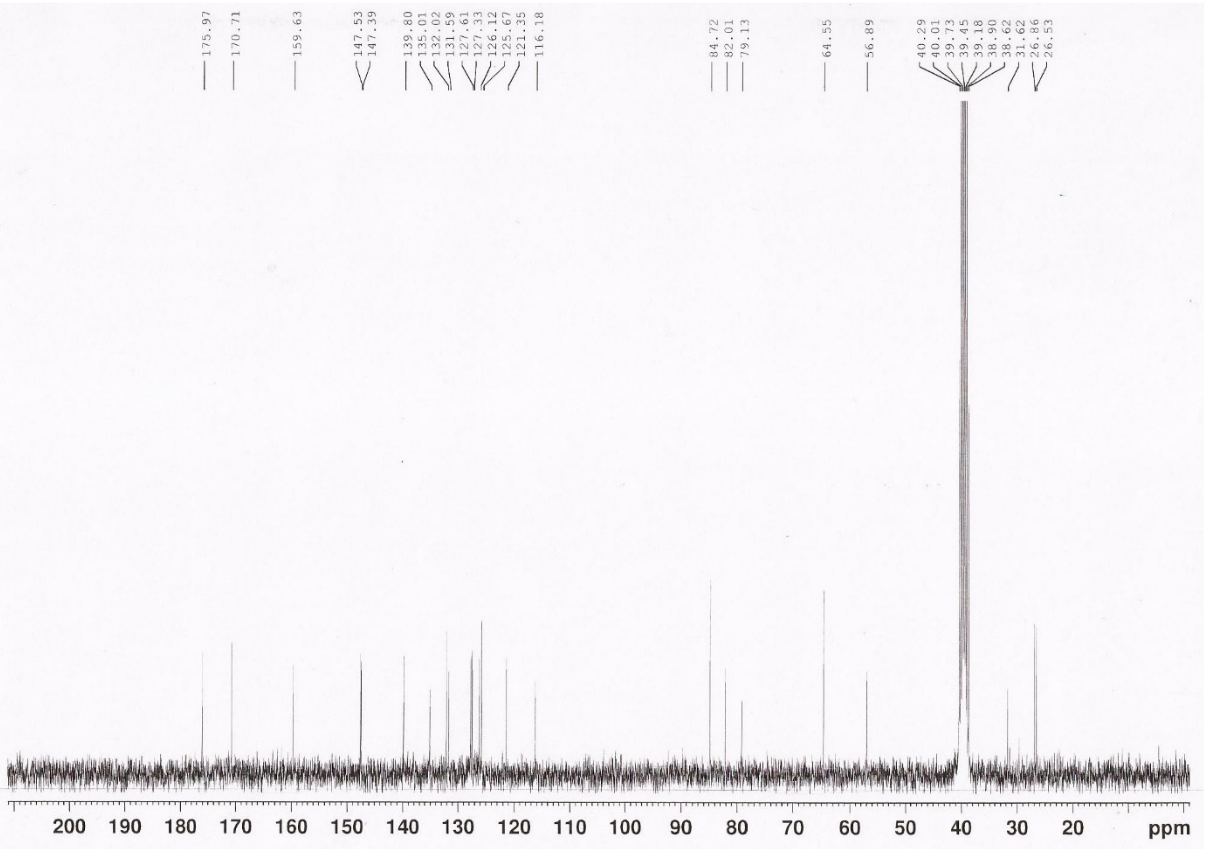

Figure S15. ^{13}C NMR spectrum of compound **3** (DMSO, 75.4 MHz).

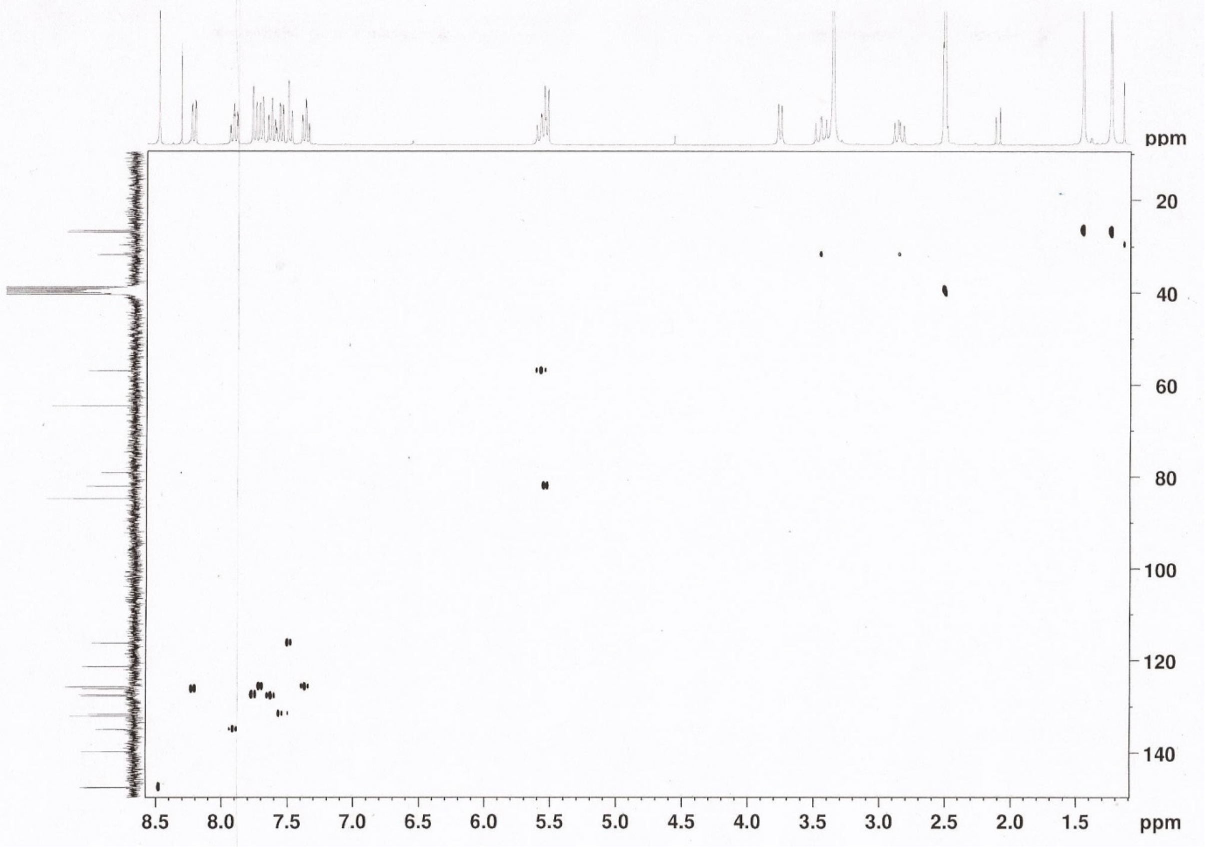

Figure S16. HSQC spectrum of compound **3** (DMSO, 300.13 MHz).

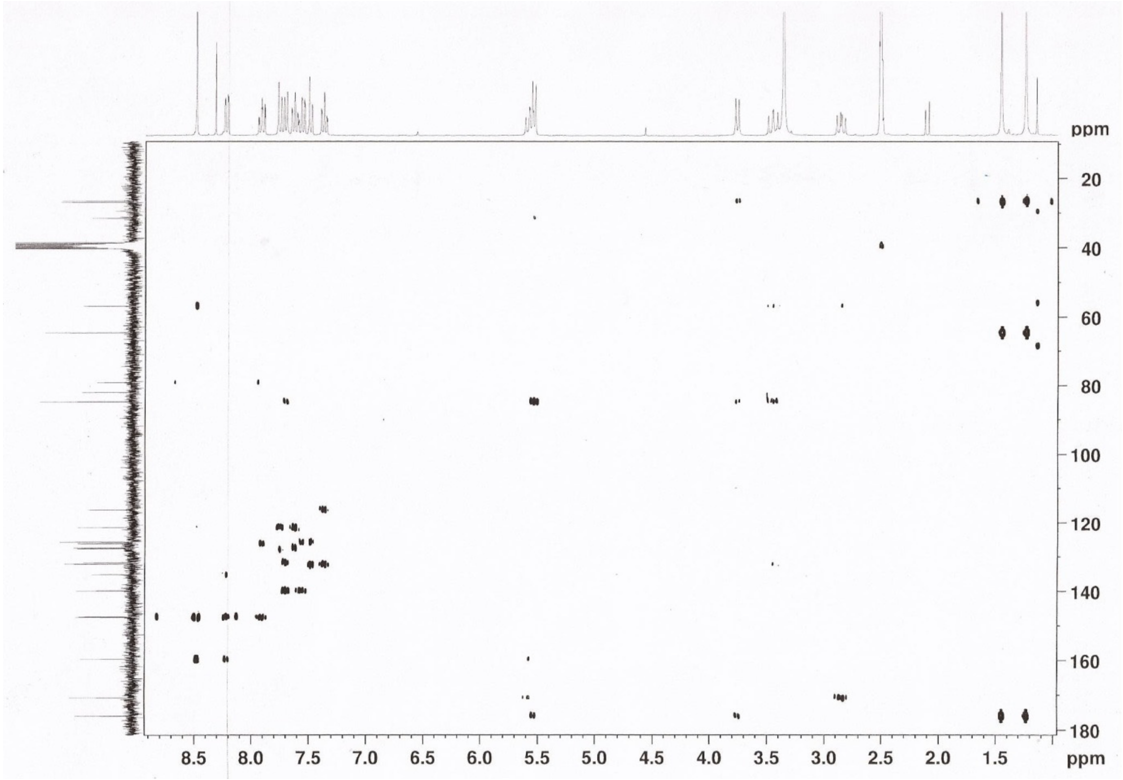

Figure S17. HMBCspectrum of compound **3** (DMSO, 300.13 MHz).

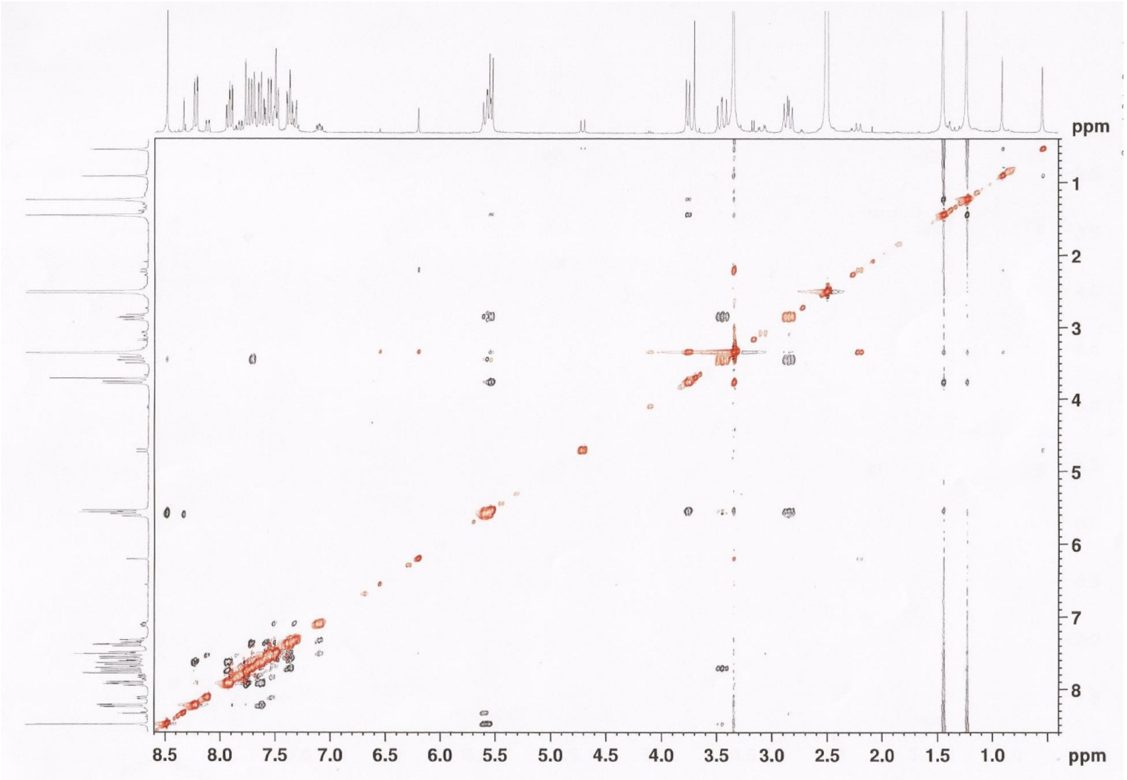

Figure S18. NOESY spectrum of compound **3** (DMSO, 300.13 MHz).

© 2015 by the authors; licensee MDPI, Basel, Switzerland. This article is an open access article distributed under the terms and conditions of the Creative Commons Attribution license (http://creativecommons.org/licenses/by/4.0/).
